# Novel *FANCA* mutation in the first fully-diagnosed patient with Fanconi anemia in Polish population – case report

**DOI:** 10.1186/s13039-020-00503-4

**Published:** 2020-08-10

**Authors:** Anna Repczynska, Agata Pastorczak, Katarzyna Babol-Pokora, Jolanta Skalska-Sadowska, Malgorzata Drozniewska, Wojciech Mlynarski, Olga Haus

**Affiliations:** 1 Department of Clinical Genetics, Collegium Medicum in Bydgoszcz, Nicolaus Copernicus University in Torun, ul. Sklodowskiej-Curie 9, 85-094 Bydgoszcz, Poland; 2 Laboratory of Immunopathology and Genetics, Department of Pediatrics, Oncology and Hematology, Medical University of Lodz, ul. Sporna 36/50, 91-738 Lodz, Poland; 3 Department of Oncology, Hematology and Pediatric Transplantology, Medical University in Poznan, ul. Szpitalna 27/33, 60-572 Poznan, Poland; 4 West Midlands Regional Genetics Laboratory, Birmingham Women’s and Children’s Hospital NHS Foundation Trust, Mindelsohn Way, B15 2TG Birmingham, UK

**Keywords:** Fanconi anemia, Pancytopenia, Chromosomal fragility, FANC genes

## Abstract

**Background:**

Fanconi anemia is a rare genetic disorder caused by mutations in genes which protein products are involved in replication, cell cycle control and DNA repair. It is characterized by congenital malformations, bone marrow failure, and high risk of cancer. The diagnosis is based on morphological and hematological abnormalities such as pancytopenia, macrocytic anaemia and progressive bone marrow failure. Genetic examination, often very complex, includes chromosomal breakage testing and mutational analysis.

**Case presentation:**

We present a child with clinical diagnosis of Fanconi anemia. Although morphological abnormalities of skin and bones were present from birth, diagnosis was only suspected at the age of 8. Chromosome breakage test in patient’s lymphocytes showed increased level of aberrations (gaps, chromatid breaks, chromosome breaks, radial figures and rearrangements) compared to control. Next generation sequencing revealed presence of two pathogenic variants in *FANCA* gene, one of which was not previously reported*.*

**Conclusions:**

The article provides additional supportive evidence that compound biallelic mutations of *FANCA* are associated with Fanconi anemia. It also illustrates the utility of combination of cytogenetic and molecular tests, together with detailed clinical evaluation in providing accurate diagnosis of Fanconi anemia. This report, to the best of our knowledge, describes the first fully diagnosed FA patient in Polish population.

## Background

Fanconi anaemia (FA) is genetically heterogenous disorder and the most common inherited bone marrow failure syndrome (IBMFS). The most frequent phenotypic features, present in about 75% of patients, include short stature, microcephaly, thumb and radial site of the limbs malformations, abnormal skin pigmentation, gastrointestinal and genitourinary defects. Progressive bone marrow failure occurs in the first decade of life, initially manifesting with leukopenia or thrombocytopenia. Acute myeloid leukemia (AML) and solid tumors of the head and neck, skin, gastrointestinal system and genitourinary system are the most common cancers occurring in patients with FA [1–3].

So far, 23 Fanconi anemia genes (FANC) have been identified: genes encoding core complex proteins – *FANCA, FANCB, FANCC, FANCE, FANCF, FANCG, FANCL* and *FANCM,* genes encoding ID2 complex proteins – *FANCD2* and *FANCI*, and downstream genes – *FANCD1(BRCA2), FANCJ(BRIP1), FANCN(PALB2), FANCO(RAD51C), FANCP(SLX4), FANCQ(ERCC4), FANCR(RAD51), FANCS(BRCA1), FANCT(UBE2T), FANCU(XRXX2), FANCV(MAD2L2/REV7), FANCW (RFWD3)* and *FANCY.* Protein products of FANC genes are involved in Fanconi anemia pathway, which regulates DNA damage repair systems [4].

On a cellular level, patients with FA are unable to process DNA lesions interfering with DNA replication. This results in increased sensitivity to DNA interstrand crosslink agents such as diepoxybutane or mitomycin C. Chromosomal breakage test is the gold standard for diagnosis of FA, but is not 100% specific. A few cases of Nijmegen breakage syndrome have been reported to give a false positive results, which was finally excluded by identification of the mutations in *NBN (NBS1)* gene. Moreover, patients with cohesinopathies – Roberts syndrome (mutated in *ESCO2*) and Warsaw Breakage Syndrome (mutated in *DDX11*) may also present positive score in the chromosome breakage test. Genes associated with FA and FA-like phenotypes encode proteins participating in DNA repair. They are also part of FA/BRCA pathway [5–8].

Cytogenetic analysis of increased levels of chromosomal breaks and radial figures after exposure to Mitomycin C (MMC) or Diepoxybutane (DEB) is recommended as a first-tier step of genetic diagnosis of Fanconi anemia. This test, although not specific for FA only, allows to differentiate FA from other chromosomal instability syndromes, such as Nijmegen syndrome, Roberts syndrome, and the Warsaw Breakage syndrome.

For molecular identification of a causative mutation confirming Fanconi anemia diagnosis, next generation sequencing (NGS) is particularly recommended.

*FANCA* gene (OMIM: 607139) is located on chromosome 16, band q24.3. The NM_000135.4 transcript consists of 43 exons [9]. Alterations of this gene are the most frequently observed gene changes in FA patients, contributing to over 60% of all FA cases [10, 11], making FA-A complementation group the most frequent in majority of countries [12]. High incidence of FA in ethnic-specific groups results from the founder effects associated with particular mutations [12].

The presence of the founder mutations in the specific FANC genes, which are common in some populations, allow to use targeted genetic tests. This applies, for example, to the Ashkenazi Jewish population with specific mutations in the *FANCC* (c.456 + 4A > T (IVS4) and *FANCD1* (c.6174delT) genes, or the Japanese population with other specific mutations: in the *FANCA* (c.2546delC or c.3720_3724del), *FANCC* (c.456 + 4A > T) and *FANCG* (c.307 + 1G > C or c.1066C > T) genes [13]. No data on the frequency of individual, recurrent mutations in FANC genes in the Polish population has been published so far.

Hematopoietic stem cell transplantation (HSCT) is the only therapy for patients with FA to cure aplastic anemia, myelodysplastic syndrome, and acute myeloid leukemia. Since this approach is associated with early and late complications, alternative treatments are still being searched for. Gene therapy approaches aiming to correct the genetic defect in the patient’s own hematopoietic stem cells remain the most promising strategy to overcome FA-associated bone marrow failure [14, 15].

Due to heterogeneous character of the *FANCA* gene variants (point mutations, insertions/deletions, splicing mutations, large intragenic deletions), the most appropriate diagnostic strategy should combine various methods [9, 12, 16]. This approach is also particularly important due to the fact that many patients harbor private mutations and that the FA phenotype overlaps with other syndromes [17].

## Case presentation

8-year old girl was referred for genetic counseling due to hematological abnormalities and birth defects. She was the only child of non-consanguineous parents, born at term after an uneventful pregnancy. Her birth weight was 2819 g. Apgar score was 9. Dysplastic left hip was noted at birth.

**Fig. 1:**
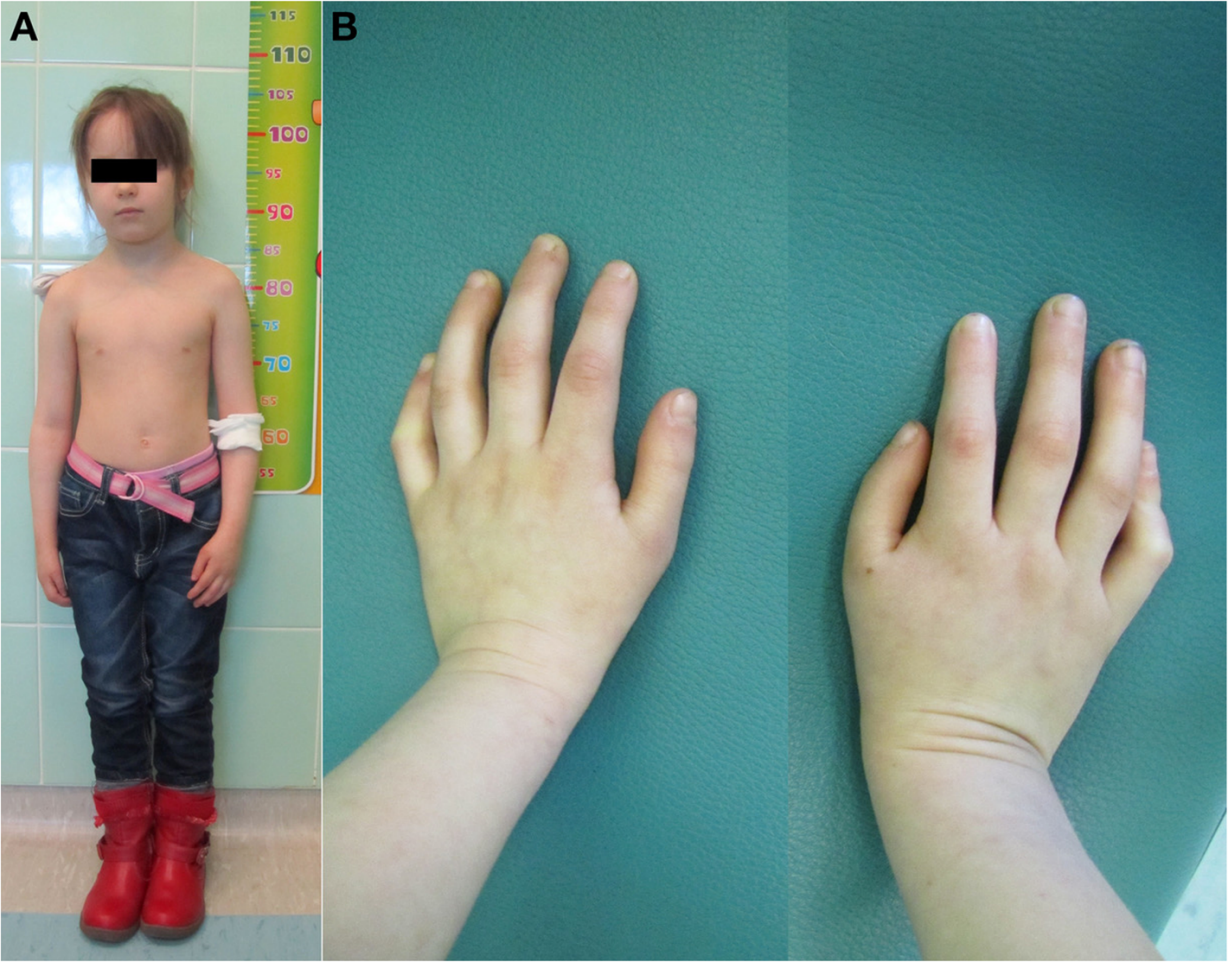
Clinical features of the patient. **a** Patient at the age of 8 – visible short stature. **b** Hypoplastic and abnormally set thumbs

Thrombocytopenia was first noted at the age of 4. Evaluation of the patient’s bone marrow trepanobioptates revealed three lineage aplasia. The examination of patient’s phenotype at the genetic counseling centre identified short stature (Fig. 1a), microcephaly, low set ears, “café-au-lait” spots, and hypoplastic thumbs (Fig. 1b). There was no intellectual disability.

## Methods

### Chromosomal breakage test

Cytogenetic studies were carried out on peripheral blood lymphocytes from parallel cultures without MMC and supplemented with MMC (50 nM, 150 nM and 300 nM). Chromosome instability data were analyzed and calculated (number of metaphases with breakage, mean chromosome breakage number per aberrant metaphase, and tri-, tetra- and multi- radials frequency) [5, 18]. A healthy control was assured.

### Molecular testing

Genomic DNA for molecular testing was extracted from peripheral blood using QIAmp DNA Mini Kit 50.

Array comparative genomic hybridization (aCGH) was performed using 8x60K Agilent SurePrint platform according to manufacturer’s protocol. The results were analyzed with Cytogenomics v.5.0 software.

Next generation sequencing (NGS) was carried out with TruSightOne Sequencing Panel, on NextSeq550 machine, Illumina in the process of 300 bp paired-end run using Mid Output Kit (Illumina). The data analyses of the target regions were performed using Burrows-Wheeler Aligner Genome Alignment Software and the GATK Variant Caller algorithms and mapped to the human genome reference sequence GRCh37/hg19 [19]. The mean region coverage depth was 126. The results were analyzed using Variant Studio v. 3.0 (Illumina) and Integrative Genomics Viewer v.2.3 [20]. The analysis focused on the following genes: *FANCA, FANCB, FANCC, BRCA2, FANCD2, FANCE, FANCF, FANCG, FANCI, BRIP1, FANCL, FANCM, PALB2, RAD51C, SLX4, ERCC4, RAD51* and *BRCA1.* The filtering criteria included coverage with at least 20 reads and a minor allele frequency (MAF) below 0.01 in 1000 Genomes and ExAC databases. The pathogenicity of the revealed changes was estimated based on standard bioinformatics tools, such as: Mutation Taster, SIFT and PolyPhen-2 and using several databases, such as ClinVar, HGMD and OMIM [21–23]. An internal database was also searched for the recurrent variants.

For verification of the variants detected in NGS we used Sanger sequencing. Standard PCR conditions were used (Supplementary Table 1) with the primers specifically designed to analyze certain mutations using NetPrimer software (Supplementary Table 2). Products were sequenced on ABI3130 4-capillary sequencer (Thermo Fisher Scientific) and the results were analyzed using Sequencher v. 5.0. (Supplementary Fig. 1).

Parental molecular follow-up studies were not performed as the patient’s parents did not consent to testing.

## Results

**Fig. 2:**
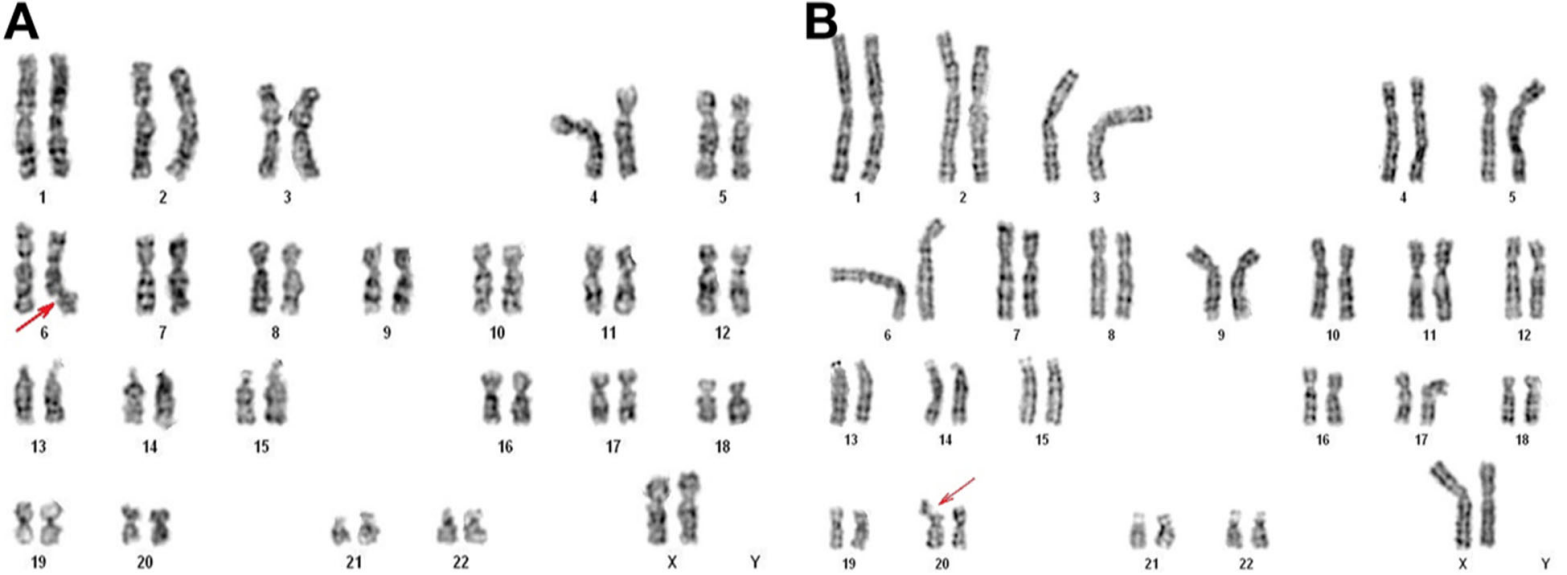
**a** and **b** Karyograms of the patient. Arrows indicate chromosomes showing spontaneous breaks

Cytogenetic GTG analysis revealed normal female karyotype. Spontaneous fragility was found in 10% of the analyzed metaphases, most frequently in chromosomes 1, 4, 6, 7, 20, 21, and X (Fig. 2 a and b).

**Fig. 3:**
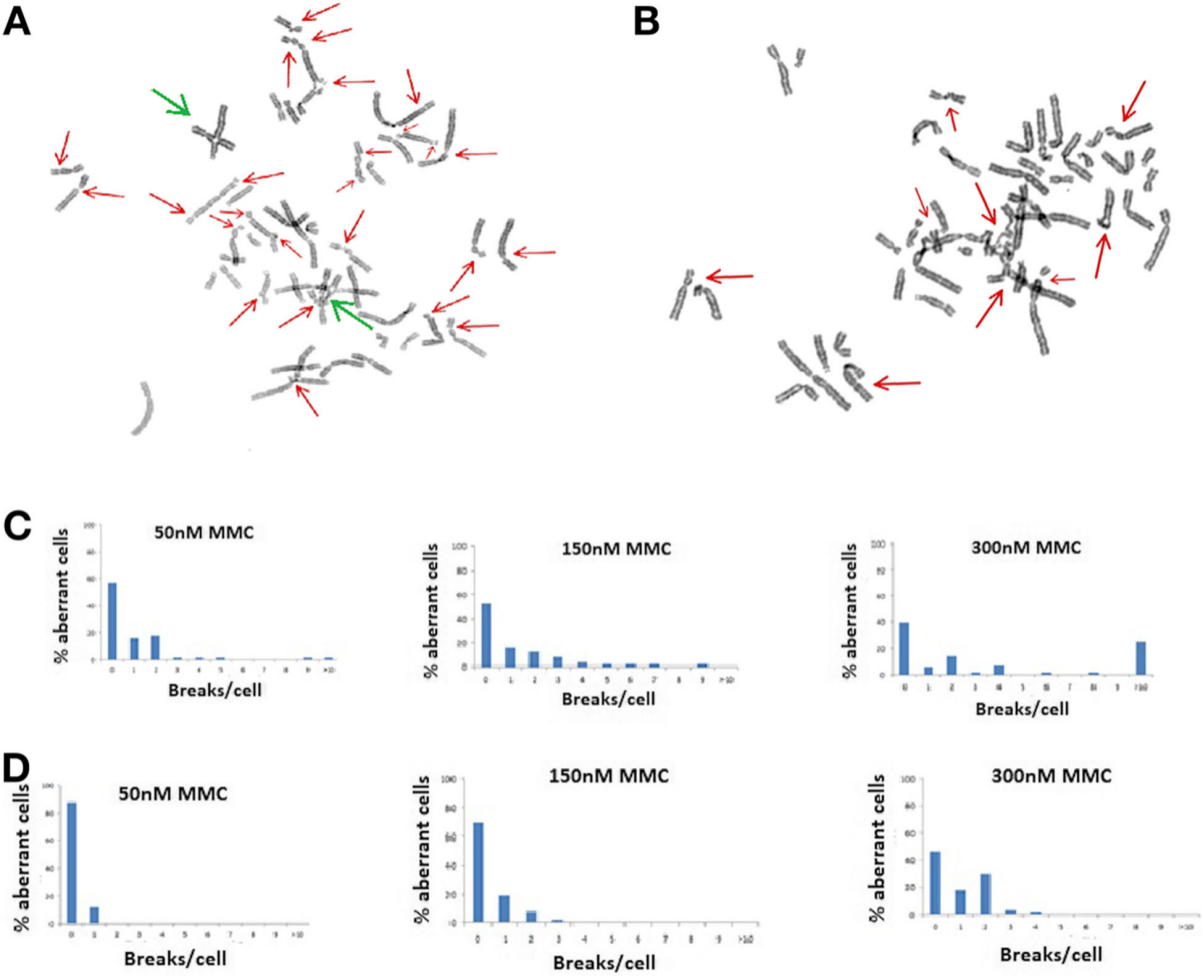
**a** and **b** Metaphase spreads in MMC test. Red arrows show gaps, chromatid breaks (chtb) and acentric fragments (ace). Green arrows show radial figures. **c** Results of chromosomal breakage MMC test in the patient. **d** Results of chromosomal breakage MMC test in healthy control

Chromosomal breakage test revealed high level of aberrations (Fig. 3 a and 3B) in patient’s lymphocytes (Fig. 3 c) compared to healthy control (Fig. 3 d).

Microarray analysis detected a female profile with no copy number imbalances that could be considered to be clinically significant.

**Fig. 4:**
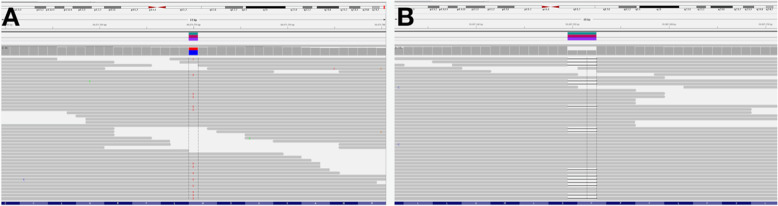
Visualization of *FANCA* sequencing reads showing c.627G>A (**a**), and c.3788_3790del (**b**)

NGS revealed presence of two pathogenic variants of the *FANCA* gene: NM_000135.4:c.627G>A, NP_000126.2:p.Trp209* and NM_000135.4:c.3788_3790del, NP_000126.2:p.Phe1263del (Fig. 4a and b, respectively). No variants of clinical significance were detected in other FANC genes.

As the parental samples have not been tested, the mode of inheritance could not have been determined.

## Discussion and conclusions

Fanconi anemia is a complex and heterogeneous disorder and the most common inherited bone marrow failure syndrome [10]. Patients struggle with progressive bone marrow failure and severe hematological complications [24]. In addition, congenital malformations are also present [25, 26].

The c.3788_3790del (p.Phe1263del) mutation, one of the mutations present in our patient, has been reported as the most frequent FA mutation worldwide. Castella et al. (2011) suggested that this results from founder character of the mutation (a common Indo-European ancestor) [12]. There are 287 entries for this mutation in the Leiden Open Variation Database [as of 21.02.2020]. All of these have been reported as germline mutations, resulting in an in-frame deletion of phenylalanine at a position that is conserved across species. This particular mutation has been reported as homozygous or compound heterozygous with other pathogenic variants in numerous FA patients [27–29]. Experimental studies have shown that this sequence change alters nuclear localization of FANCA protein and impairs the function of FANCA in vitro, and therefore this variant has been classified as pathogenic [12].

The second mutation found in our patient, c.627G>A (p.Trp209*), has not been reported before. It has neither been found on ExAC nor 1000G. It suggests that this particular mutation is a private one for our patient.

Fanconi anemia presents with wide spectrum of phenotypes, affecting multiple systems [30]. Although some features, including short stature, café au lait spots are common in the majority of the patients, it is difficult to establish a ‘typical’ phenotype for FA. It has been reported that classical and most frequently occurring congenital abnormalities are consistent with those seen in the VACTERL-H association (V – vertebral, A – anal, C – cardiac, T – tracheo-esophageal fistula, E – esophageal atresia, R – renal, L – upper limb and H – hydrocephalus) [30, 31]. The association is defined based on harboring at least three of the anomalies mentioned above [26]. Recently, Fiesco-Roa et al. described other abnormalities common in FA patients, but not included in the VACTERL-H. They have been grouped as PHENOS (P – pigmentation, H – small head, E – small eyes, N – nervous system, O – otology, S – short stature). According to this study, patients with FA with VACTERL-H characteristics also revealed at least four out of six PHENOS features [30]. The patient reported by us had only a couple of phenotypic features of FA – short stature and hypoplastic and abnormally set thumbs. Her intellectual level was normal. However, she fits into the spectrum of FA phenotypes. There is an important phenotypic heterogeneity in this disease.

Steinberg-Shemer et al. (2019) analyzed FANC genes by Sanger sequencing in 94 FA patients in order to find a genotype-phenotype correlation. They reported no association between the affected FA gene and survival. No specific gene has been reported in association with bone marrow failure. Patients with *FANCD1* and *FANCJ* mutations were significantly shorter compared to others. Downstream genes mutations were found to be more frequent in patients with skull, central nervous system and genitourinary anomalies, in comparison to patients with core complex genes mutations [32].

It has also been reported that patients with *FANCA* mutations developed MDS/leukemia at a significantly older age opposed to non-*FANCA* mutations patients. The authors also report on significant correlation between *FANCA* mutation type and congenital anomalies: short stature was present more often in patients with deletions than with nonsense mutations. Splice-site mutations positively correlated with developmental delay and central nervous system anomalies, whereas missense mutations were present less often in association with congenital heart disease [32].

Genetic study should be performed, if possible, in all patients with FA suspicion, their siblings and parents. It is important to stress that identification of FANC gene causative mutations in any clinically suspected child not only supports clinical diagnosis enabling appropriate treatment, including less genotoxic medical procedures, and mild conditioning regimens before HSCT, but also allows appropriate genetic counseling for the whole family. It also facilitates accurate and targeted molecular studies, i.e. by non-invasive prenatal diagnosis (NIPD).

The parents of our patient were not tested, therefore their carrier status, if any, is unknown.

Their genetic risk of having future children with FA is 25%, under the assumption that each of them is a heterozygous carrier of one of the mutations found in their daughter. Therefore, a prenatal diagnosis should be offered to the mother of our patient at each subsequent pregnancy.

## Supplementary information

**Additional file 1: Figure S1.** Sanger sequencing chromatograms showing the *FANCA* mutations detected in the reported patient: c.627G > A (A) and c.3788_3790del (B).

**Additional file 2: Table S1.** The sequences of primers used for verification of pathogenic *FANCA* variants detected in the reported patient using Sanger sequencing.

**Additional file 3: Table S2.** Characteristics of the pathogenic *FANCA* variants identified in the reported patient.

## Data Availability

Complete datasets used and analysed during the current study are available from the corresponding author on reasonable request.

## Abbreviations

IBMFS: Inherited Bone Marrow Failure Syndrome
AML: Acute Myeloid Leukemia
MMC: Mitomycin C
FA: Fanconi Anemia
DEB: Diepoxybutane
NGS: Next Generation Sequencing
HSCT: Hematopoietic Stem Cell Transplantation
aCGH: Array Comparative Genomic Hybridisation
VACTERL-H: Association of features including Vertebral, Anal, Cardiac, Tracheo-esophageal fistula, Esophageal atresia, Renal, upper Limb and Hydrocephalus
PHENOS: Association of abnormalities including Pigmentation, small Head, small Eyes, Nervous system, Otology, Short stature
