# Supplementary figures and images for: Novel FANCA mutation in the first fully-diagnosed patient with Fanconi anemia in Polish population – case report
Source: Mol Cytogenet. 2020 Aug 10;13:33. doi: 10.1186/s13039-020-00503-4 (PMC7418427; doi:10.1186/s13039-020-00503-4)

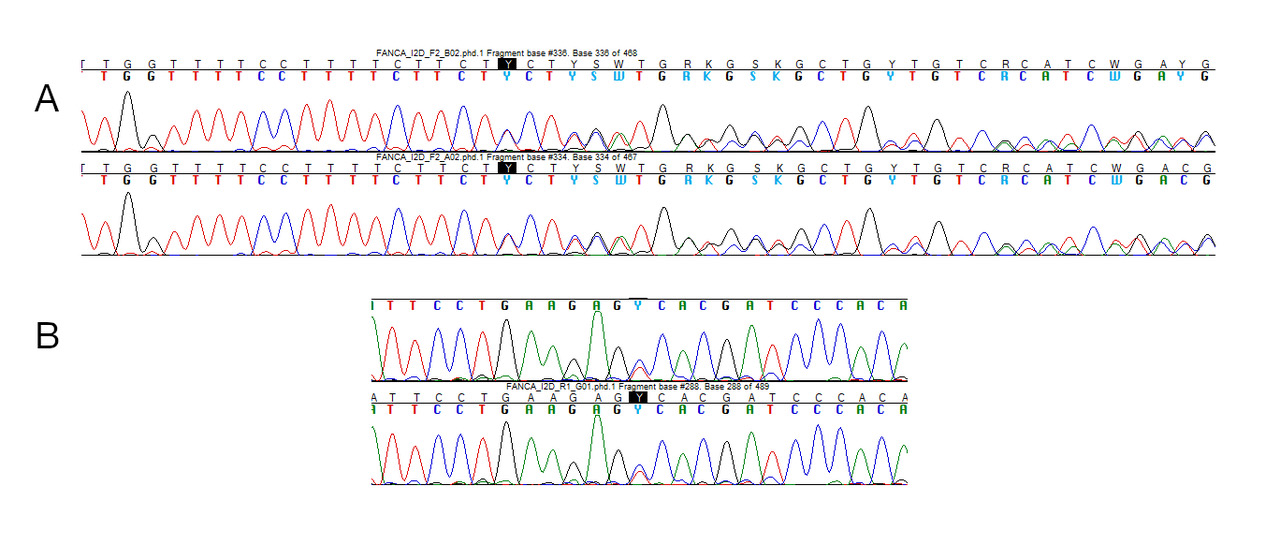

Supplement: Supplementary file 1 — Additional file 1: Figure S1. Sanger sequencing chromatograms showing the FANCA mutations detected in the reported patient: c.627G > A (A) and c.3788_3790del (B). [file 13039_2020_503_MOESM1_ESM.jpeg]
