# Supplementary material for: Novel FANCA mutation in the first fully-diagnosed patient with Fanconi anemia in Polish population – case report
Source: Mol Cytogenet. 2020 Aug 10;13:33. doi: 10.1186/s13039-020-00503-4 (PMC7418427; doi:10.1186/s13039-020-00503-4)
Supplement: Supplementary file 2 — Additional file 2: Table S1. The sequences of primers used for verification of pathogenic FANCA variants detected in the reported patient using Sanger sequencing. [file 13039_2020_503_MOESM2_ESM.doc]

Supplementary Table 1. The sequences of primers used for verification of pathogenic *FANCA* variants detected in our patient using Sanger sequencing.

|  |  |  | Tm |
| --- | --- | --- | --- |
| c.627G>A | Forward | 5' GGCTCAACTCAATCCCCTGATG 3' | 57.0°C |
| c.627G>A | Reverse | 5' GGAAAGGTGAATGGAAACACTTAAACTC 3' | 57.0°C |
| c.3788_3790del | Forward | 5' TTTACAAACACTTCTGACAGGAGGC 3' | 57.0°C |
| c.3788_3790del | Reverse | 5' AGTTCTCACTCACACTTCCGCAAAC 3' | 57.0°C |
