# Supplementary material for: Novel FANCA mutation in the first fully-diagnosed patient with Fanconi anemia in Polish population – case report
Source: Mol Cytogenet. 2020 Aug 10;13:33. doi: 10.1186/s13039-020-00503-4 (PMC7418427; doi:10.1186/s13039-020-00503-4)
Supplement: Supplementary file 3 — Additional file 3: Table S2. Characteristics of the pathogenic FANCA variants identified in the reported patient. [file 13039_2020_503_MOESM3_ESM.doc]

Supplementary Table 2. Characteristics of the pathogenic *FANCA* variants identified in the reported patient.

| Gene | FANCA | FANCA |
| --- | --- | --- |
| Chromosome | 16 | 16 |
| Cytoband | 16q24.3 | 16q24.3 |
| Position | 89871770 | 89807250 |
| Ref sequence | C | AGA |
| Alt sequence | T |  |
| HGVS | NM_000135.4:c.627G>A | NM_000135.4:c.3788_3790del |
| Protein | NP_000126.2:p.Trp209* | NP_000126.2:p.Phe1263del |
| Consequence | Stop gained | Inframe deletion |
| Frequency (GnomAD_exome) | No data available | 0.0000997 |
| GERP# score | 4.9 | 2.61 |
| DANN* score | 0.9938 | No data available |
| MutationTaster score | 0.81 | No data available |
| ACMG classification | Pathogenic | Likely pathogenic |
| Total Read Depth | 76 | 104 |

# Genomic Evolutionary Rate Profiling – is a conservation score calculated by quantifying substitution deficits across multiple alignments of orthologues using the genomes of 33 mammals. It ranges from -12.3 to 6.17, with 6.17 being the most conserved.

* DANN is a pathogenicity scoring methodology which based on deep neural networks. The value range is 0 to 1, with 1 given to the variants predicted to be the most damaging.
